# A redetermination at low temperature of the structure of triethyl­ammonium bromide

**DOI:** 10.1107/S1600536808034843

**Published:** 2008-10-31

**Authors:** Natasha H. Munro, Lyall R. Hanton

**Affiliations:** a Department of Chemistry, University of Otago, PO Box 56, Dunedin, New Zealand

## Abstract

The structure of the title compound, C_6_H_16_N^+^·Br^−^, was determined at low temperature and the cell dimensions were comparable to those reported for room-temperature studies [James, Cameron, Knop, Newman & Falp, (1985). *Can. J. Chem.* **63**, 1750–1758]. Initial analysis of the data led to the assignment of *P*3_1_ *c* as the space group rather than *P*6_3_ *mc* as reported for the room-temperature structure. Careful examination of the appropriate |*F*_o_| values in the low-temperature data showed that the equalities |*F*(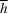 *kl*)| = |*F*(*h* 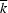 *l*)| and |F(*hkl*)| = |*F*(*hk* 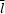)| did not hold at low temperature, confirming *P*3_1_c as the appropriate choice of space group. As a consequence of this choice, the N atom sat on a threefold axis and the ethyl arms were not disordered as observed at room temperature. The crystal studied was an inversion twin with a 0.68 (3):0.32 (3) domain ratio.

## Related literature

For related structures, see: James *et al.* (1985 ▶). For the preparation, see: Lecolley *et al.* (2004 ▶).
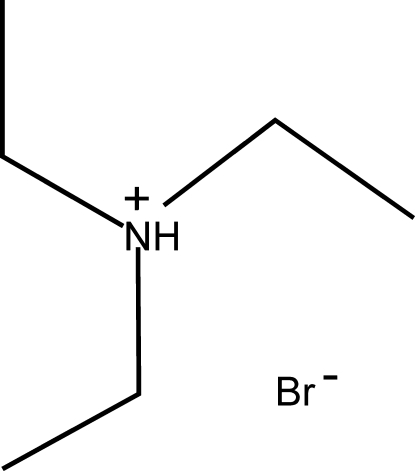


## Experimental

### 

#### Crystal data

C_6_H_16_N^+^·Br^−^
*M* *_r_* = 182.10
Trigonal, 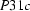

*a* = 8.3589 (2) Å
*c* = 7.3125 (2) Å
*V* = 442.48 (1) Å^3^
*Z* = 2
Mo *K*α radiation
μ = 4.56 mm^−1^
*T* = 90 (2) K
0.27 × 0.11 × 0.10 mm

#### Data collection

Bruker APEXII CCD area-detector diffractometer
Absorption correction: multi-scan (*SADABS*; Bruker, 2004 ▶) *T*_min_ = 0.450, *T*_max_ = 0.632
8583 measured reflections
555 independent reflections
550 reflections with *I* > 2σ(*I*)
*R*_int_ = 0.026

#### Refinement

*R*[*F* ^2^ > 2σ(*F* ^2^)] = 0.020
*wR*(*F* ^2^) = 0.058
*S* = 1.24
555 reflections
23 parameters
1 restraint
H-atom parameters constrained
Δρ_max_ = 0.39 e Å^−3^
Δρ_min_ = −0.39 e Å^−3^
Absolute structure: Flack (1983 ▶), 273 Friedel pairs
Flack parameter: 0.32 (3)

### 

Data collection: *APEX2* (Bruker, 2006 ▶); cell refinement: *APEX2* and *SAINT* (Bruker, 2006 ▶); data reduction: *SAINT*; program(s) used to solve structure: *SIR97* (Altomare *et al.*, 1993 ▶); program(s) used to refine structure: *SHELXL97* (Sheldrick, 2008 ▶); molecular graphics: *SHELXTL* (Sheldrick, 2008 ▶); software used to prepare material for publication: *SHELXTL*.

## Supplementary Material

Crystal structure: contains datablocks global, I. DOI: 10.1107/S1600536808034843/pv2113sup1.cif

Structure factors: contains datablocks I. DOI: 10.1107/S1600536808034843/pv2113Isup2.hkl

Additional supplementary materials:  crystallographic information; 3D view; checkCIF report

## Figures and Tables

**Fig. 1.**
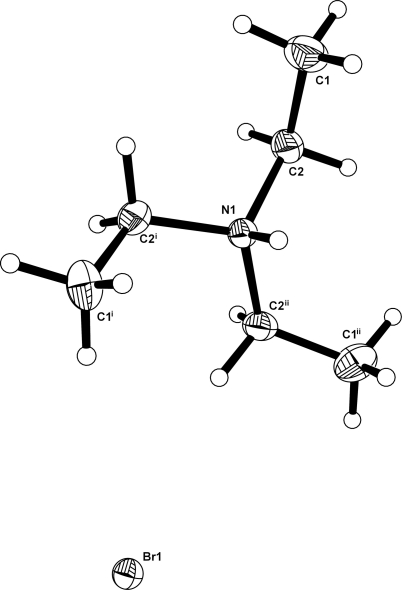
A view of the molecule of (I) showing the atom numbering with displacement ellipsoids drawn at the 50% probability level. Symmetry codes: (i) -x+y, -x+1, z; (ii) -y+1, x-y+1, z.

**Fig. 2.**
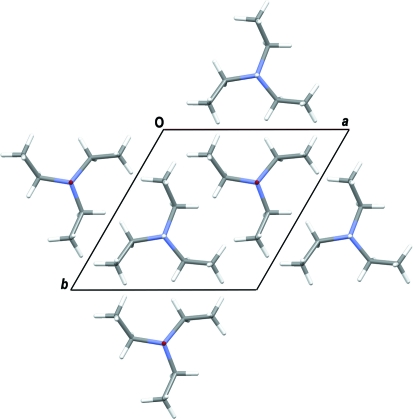
Packing diagram of (I) in the ab plane.

## supplementary crystallographic information

### Comment

The title compound, (I), was isolated as a by-product in a reaction to form (2,5-oxo-1-pyrrolidyl)oxy-2-bromo-2-methylpropionate (Lecolley *et al.*, 2004). A view of the structure of (I) is presented in Fig. 1. The crystal structures of (I) and the other halide analogues at ambient temperature have previously been described by James *et al.* (1985). Unlike previous work, analysis of our low-temperature data showed that (I) crystallized in the space group P3_1_c with the ethyl chains in fixed locations. The e.s.d.'s of the positional parameters and the *R* factors were significantly lower than those reported for the room temperature structure. The packing of (I) (Fig. 2) at low temperature is very similar to that of the room temperature disordered structure. James *et al.* (1985) also analysed the IR spectra of these compounds in some detail.

### Experimental

The title compound, (I), was prepared as a by-product in a reaction to form (2,5-oxo-1-pyrrolidyl)oxy-2-bromo-2-methylpropionate by the method of Lecolley *et al.* (2004). X-Ray quality crystals were grown by the slow evaporation of an acetonitrile solution.

### Refinement

All H-atoms bound to carbon were refined using a riding model with d(C—H) = 0.96 Å, *U*_iso_=1.5*U*_eq_ (C) for the methyl CH H atoms and d(C—H) = 0.97 Å, *U*_iso_=1.2*U*_eq_ (C) for the methylene CH H atoms. The H-atom bound to nitrogen was refined using a riding model with d(N—H) = 0.87 Å, *U*_iso_=1.2*U*_eq_ (N).

### Crystal data

**Table:**

C_6_H_16_N^+^·Br^−^	*D*_x_ = 1.367 Mg m^−^^3^
*M**_r_* = 182.10	Mo *K*α radiation, λ = 0.71073 Å
Trigonal, *P*31*c*	Cell parameters from 7729 reflections
Hall symbol: P 3 -2c	θ = 2.8–27.5°
*a* = 8.3589 (2) Å	µ = 4.56 mm^−^^1^
*c* = 7.3125 (2) Å	*T* = 90 K
*V* = 442.48 (1) Å^3^	Rod, colourless
*Z* = 2	0.27 × 0.11 × 0.10 mm
*F*(000) = 188	

### Data collection

**Table:**

Bruker APEXII CCD area-detector diffractometer	555 independent reflections
Radiation source: fine-focus sealed tube	550 reflections with *I* > 2σ(*I*)
graphite	*R*_int_ = 0.026
φ and ω scans	θ_max_ = 25.5°, θ_min_ = 4.0°
Absorption correction: multi-scan (SADABS; Bruker, 2004)	*h* = −10→10
*T*_min_ = 0.450, *T*_max_ = 0.633	*k* = −10→10
8583 measured reflections	*l* = −8→8

### Refinement

**Table:**

Refinement on *F*^2^	Secondary atom site location: difference Fourier map
Least-squares matrix: full	Hydrogen site location: inferred from neighbouring sites
*R*[*F*^2^ > 2σ(*F*^2^)] = 0.020	H-atom parameters constrained
*wR*(*F*^2^) = 0.058	*w* = 1/[σ^2^(*F*_o_^2^) + (0.0371*P*)^2^ + 0.4873*P*] where *P* = (*F*_o_^2^ + 2*F*_c_^2^)/3
*S* = 1.24	(Δ/σ)_max_ < 0.001
555 reflections	Δρ_max_ = 0.39 e Å^−^^3^
23 parameters	Δρ_min_ = −0.39 e Å^−^^3^
1 restraint	Absolute structure: Flack (1983), 273 Friedel pairs
Primary atom site location: structure-invariant direct methods	Flack parameter: 0.32 (3)

### Special details

**Table:**

Geometry. All e.s.d.'s (except the e.s.d. in the dihedral angle between two l.s. planes) are estimated using the full covariance matrix. The cell e.s.d.'s are taken into account individually in the estimation of e.s.d.'s in distances, angles and torsion angles; correlations between e.s.d.'s in cell parameters are only used when they are defined by crystal symmetry. An approximate (isotropic) treatment of cell e.s.d.'s is used for estimating e.s.d.'s involving l.s. planes.
Refinement. Refinement of *F*^2^ against ALL reflections. The weighted *R*-factor *wR* and goodness of fit *S* are based on *F*^2^, conventional *R*-factors *R* are based on *F*, with *F* set to zero for negative *F*^2^. The threshold expression of *F*^2^ > σ(*F*^2^) is used only for calculating *R*-factors(gt) *etc*. and is not relevant to the choice of reflections for refinement. *R*-factors based on *F*^2^ are statistically about twice as large as those based on *F*, and *R*- factors based on ALL data will be even larger. The crystal studied was an inversion twin with a 0.68 (3);0.32 (3) domain ratio.

### Fractional atomic coordinates and isotropic or equivalent isotropic displacement parameters (Å^2^)

**Table:**

	*x*	*y*	*z*	*U*_iso_*/*U*_eq_	
C1	0.1624 (6)	0.8395 (6)	0.4111 (5)	0.0323 (9)	
H1A	0.1536	0.8181	0.2815	0.048*	
H1B	0.2690	0.9571	0.4373	0.048*	
H1C	0.0533	0.8389	0.4534	0.048*	
N1	0.3333	0.6667	0.4505 (6)	0.0168 (12)	
H1	0.3333	0.6667	0.3260	0.020*	
C2	0.1789 (5)	0.6982 (5)	0.5011 (5)	0.0232 (7)	
H2A	0.0644	0.5830	0.4820	0.028*	
H2B	0.1884	0.7240	0.6312	0.028*	
Br1	0.6667	0.3333	0.5017	0.01786 (16)	

### Atomic displacement parameters (Å^2^)

**Table:**

	*U*^11^	*U*^22^	*U*^33^	*U*^12^	*U*^13^	*U*^23^
C1	0.034 (2)	0.038 (2)	0.035 (2)	0.026 (2)	−0.0004 (16)	−0.0026 (17)
N1	0.0162 (14)	0.0162 (14)	0.018 (3)	0.0081 (7)	0.000	0.000
C2	0.0168 (15)	0.0228 (14)	0.0293 (17)	0.0095 (12)	0.0004 (14)	0.0001 (16)
Br1	0.01867 (19)	0.01867 (19)	0.0162 (2)	0.00933 (9)	0.000	0.000

### Geometric parameters (Å, °)

**Table:**

C1—C2	1.418 (5)	N1—C2^i^	1.488 (4)
C1—H1A	0.9600	N1—C2^ii^	1.488 (4)
C1—H1B	0.9600	N1—H1	0.9100
C1—H1C	0.9600	C2—H2A	0.9700
N1—C2	1.488 (4)	C2—H2B	0.9700
			
C2—C1—H1A	109.5	C2—N1—H1	104.4
C2—C1—H1B	109.5	C2^i^—N1—H1	104.4
H1A—C1—H1B	109.5	C2^ii^—N1—H1	104.4
C2—C1—H1C	109.5	C1—C2—N1	119.1 (3)
H1A—C1—H1C	109.5	C1—C2—H2A	107.5
H1B—C1—H1C	109.5	N1—C2—H2A	107.5
C2—N1—C2^i^	114.03 (18)	C1—C2—H2B	107.5
C2—N1—C2^ii^	114.03 (18)	N1—C2—H2B	107.5
C2^i^—N1—C2^ii^	114.03 (18)	H2A—C2—H2B	107.0

Symmetry codes: (i) −*x*+*y*, −*x*+1, *z*; (ii) −*y*+1, *x*−*y*+1, *z*.
